# Decoding the enigmatic role of T-cadherin in tumor angiogenesis

**DOI:** 10.3389/fimmu.2025.1564130

**Published:** 2025-03-31

**Authors:** Yiyang Wen, Li Ma, Yuanyuan Liu, Huabao Xiong, Dongmei Shi

**Affiliations:** ^1^ The Laboratory of Medical Mycology, Jining No.1 People’s Hospital, Jining, Shandong, China; ^2^ Department of Pathology, Jining No.1 People’s Hospital, Jining, Shandong, China; ^3^ The Second Clinical Medical College, Shandong University of Traditional Chinese Medicine, Jinan, Shandong, China; ^4^ Institute of Immunology and Molecular Medicine, Jining Medical University, Jining, Shandong, China; ^5^ Department of Dermatology, Jining No.1 People’s Hospital, Jining, Shandong, China

**Keywords:** T-cadherin, endothelial cells, tumor angiogenesis, tumor, VEGF

## Abstract

The cadherin family, which includes T-cadherin, plays a significant role in angiogenesis, a critical process involved in tumor growth, metastasis, and recurrence. T-cadherin is extensively expressed in both normal and tumor vascular tissues and has been shown to facilitate the proliferation and migration of vascular cells in some studies. However, T-cadherin also exerts inhibitory effects on angiogenesis in various tumor tissues. The functional role of T-cadherin may vary depending on the tumor type and the interaction between tumor cells and vascular cells, suggesting that it acts as a modulator rather than a primary driver of angiogenesis. Additionally, T-cadherin exhibits distinct characteristics depending on the tumor microenvironment. This review provides an overview of recent research on the role of T-cadherin in tumor angiogenesis and discusses its potential as a diagnostic or therapeutic marker in the field of tumor biology.

## 1 Introduction

Tumor progress relies on the expansion of blood vessels to supply essential nutrients and oxygen to malignant cells, resulting in an aberrant tumor microenvironment. This process promotes tumor proliferation, invasion, and metastasis (1–3). Intratumor vessels commonly exhibit structural and functional abnormalities, including disorganized vascular structure, disruption of vascular endothelial cell (EC) junctions, loss of pericyte coverage and an irregular or deficient basal membrane (4, 5). These anomalies impact tumor growth and dissemination, leading to alterations in the tumor microenvironment conducive to tumor progression (6, 7).

T-cadherin, also known as H-cadherin or cadherin-13, was first identified in 1991 (8). T-cadherin has a distinct structure compared to classical cadherins, lacking transmembrane and cytoplasmic domains, but is membrane-bound due to a covalently attached glycosylphosphatidylinisitol (GPI) anchor (9). Unlike other cadherins, T-cadherin does not function primarily as an intercellular adhesion molecule due to the absence of an intracellular domain. Instead, it is believed to be involved in particular intracellular signal pathways (10). Increasing evidence shows that T-cadherin plays an essential role in regulating not only the proliferation, invasion and metastasis of the tumor cell but also in tumor angiogenesis across various cancers including lung, ovarian, esophageal, bladder, cervical and prostate carcinoma (11, 12). However, there is still controversy surrounding the specific functions and underlying mechanisms of T-cadherin in tumor angiogenesis. This review offers a historical overview of T-cadherin research and highlights its significance in tumor angiogenesis.

## 2 Atypical structure of T-cadherin

Human CDH13 (Truncated cadherin, T-cadherin) is located on chromosome 16q24, adjacent to CDH5 (Vascular endothelial cadherin, VE-cadherin), CDH1 (Epithelial cadherin, E-cadherin), CDH3 (Placental cadherin, P-cadherin), CDH8 (Cadherin-8) and CDH11 (Osteoblast cadherin, OB-cadherin), and it is highly conserved across other species in evolution (13). CDH13 gene contains 14 exons, encoding a cDNA sequence of 2142 bps that can be translated into a protein consisting of 713 amino acids (14).

**Figure 1:**
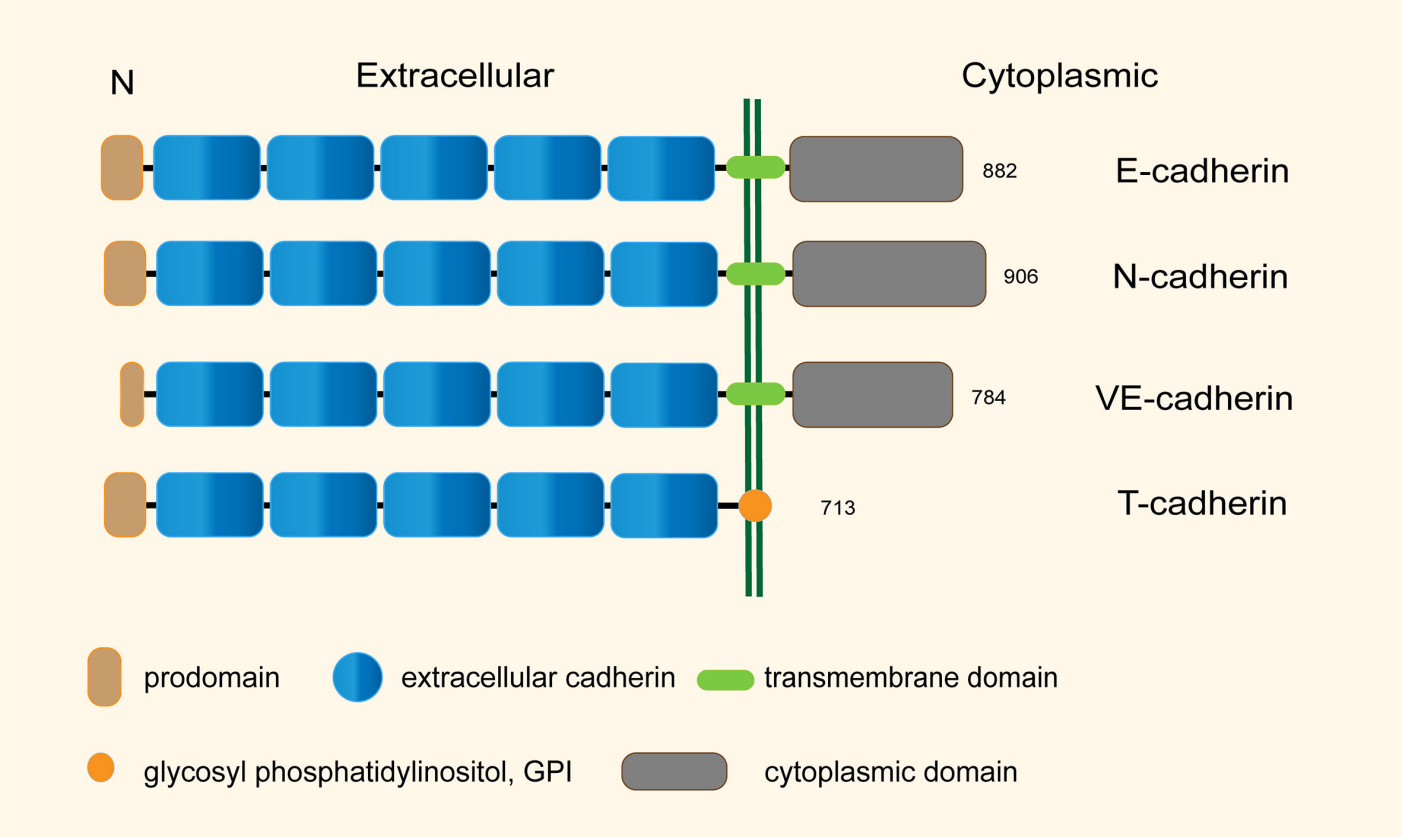
Structural characteristics of the representative human cadherins (total sizes in amino acid residues are shown).

Classical cadherins contain extracellular cadherin repeats, a single transmembrane domain, and a cytoplasmic domain with highly conserved binding sites for downstream catenins, such as p120−catenin and β−catenin, which in turn bind to α-catenin, polymerizing actin microfilaments and maintaining the stability of cytoskeleton. In contrast, T-cadherin lacks transmembrane and cytoplasmic domains and is inserted to the membrane through glycosyl phosphatidylinositol (GPI) attached to the apical aspect plasma membrane (**Figure 1**) (15), but lacks key amino acids for the adhesive functions. Notably, T-cadherin does not have the canonical strand-exchange dimer and lacks the conserved HisAlaVal motif responsible for homophilic adhesion. These differences indicate that the adhesive mechanisms of T-cadherin may be distinct from classical cadherins (16–18).

## 3 T-cadherin expression and function in vascular tissues

### 3.1 Expression pattern of T-cadherin in vascular tissues and cells

Early immunohistochemical studies have confirmed that T-cadherin is highly expressed in all cardiovascular tissues, including heart, aorta, arteries, post cava and capillaries (19). It is especially found in endothelial cells (ECs), smooth muscle cells (SMCs) and pericytes. In pathological conditions like atherosclerosis, restenosis after balloon angioplasty and tumor angiogenesis, T-cadherin is upregulated contributing to excessive migration, proliferation, and phenotypic modulation of vascular cells (20–22).

### 3.2 Proliferative effects of T-cadherin on vascular cells

T-cadherin has been shown to promote proliferation in ECs and SMCs. It activates the PI3K/Akt/mTOR pathway and inhibits the p38MAPK pathway, protecting ECs from stress-induced apoptosis (23). T-cadherin also affects cell cycle progression, with an increased expression during early S-phase and promoting proliferation in both ECs and SMCs (24). Additionally, T-cadherin influences the expression of β-catenin and ILK, leading to cell proliferation and protection against apoptosis (25). However, T-cadherin knockdown strongly inhibited proliferation of pericytes (26).

### 3.3 Regulation of T-cadherin in vascular cell plasticity and motility

Although the stable extracellular structural domain 1 (EC1) is not favorable for homologous binding, homophilic inhibition by recombinant T-cadherin protein against the T-cadherin EC1 domain significantly decreased the adhesion of SMCs and HUVECs (27). At the same time, adenovirus mediated overexpression of T-cadherin can increase detachment and migration of HUVECs, suggesting an anti-adhesive role of T-cadherin for vascular cells. Moreover, T-cadherin could promote SMCs to dedifferentiate upon GSK3β inactivation, which is characterized by acquisition of synthetic, migratory and proliferative properties in response to vascular injury or the presence of atherosclerosis (28). These observations were then confirmed in the subsequent study by using 2D-monolayer and 3D-spheroid migration models (29). The studies collectively demonstrate the function of T-cadherin in promoting vascular cell migration and inhibiting adhesion.

Another study reported that homophilic activation of T-cadherin in HUVECs induced morphological changes toward promigratory phenotype via RhoA/ROCK and Rac pathways and changed adhesion and polarization of the ECs (30). In the initial stage of angiogenesis, the contraction, stretching and remodeling of ECs has been shown to depend critically on RhoA/ROCK activation (31). The Rac activation is necessary for VEGF-induced migration, lamellipodia formation, and recruiting high-affinity integrins to lamellipodia, inducing formation of dynamic cellular protrusions and capillary structures at the leading edges of polarized cells during angiogenesis (32, 33). However, E. V. Semina et al. found that the expression of T-cadherin can lead to an activation of Rac1 and Cdc42, but have no effects on the RhoA signaling pathway (10). The difference in T-cadherin overexpression induced GTPases activation could possibly be related with different experimental conditions or the cell adhesion state.

### 3.4 Degradation of VE-cadherin in barrier function

T-cadherin overexpression leads to the degradation of VE-cadherin in lysosomes, disrupting endothelial barrier function and increasing permeability (34–36). T-cadherin’s involvement in GTPases-mediated signaling pathways affects actin stress fiber formation and microtubule polymerization, leading to decreased permeability of the endothelial monolayer (10, 37). T-cadherin also plays a significant role in regulating endothelial barrier function in response to serum and thrombin (38).

### 3.5 T-cadherin in capillary initiation

**Figure 2:**
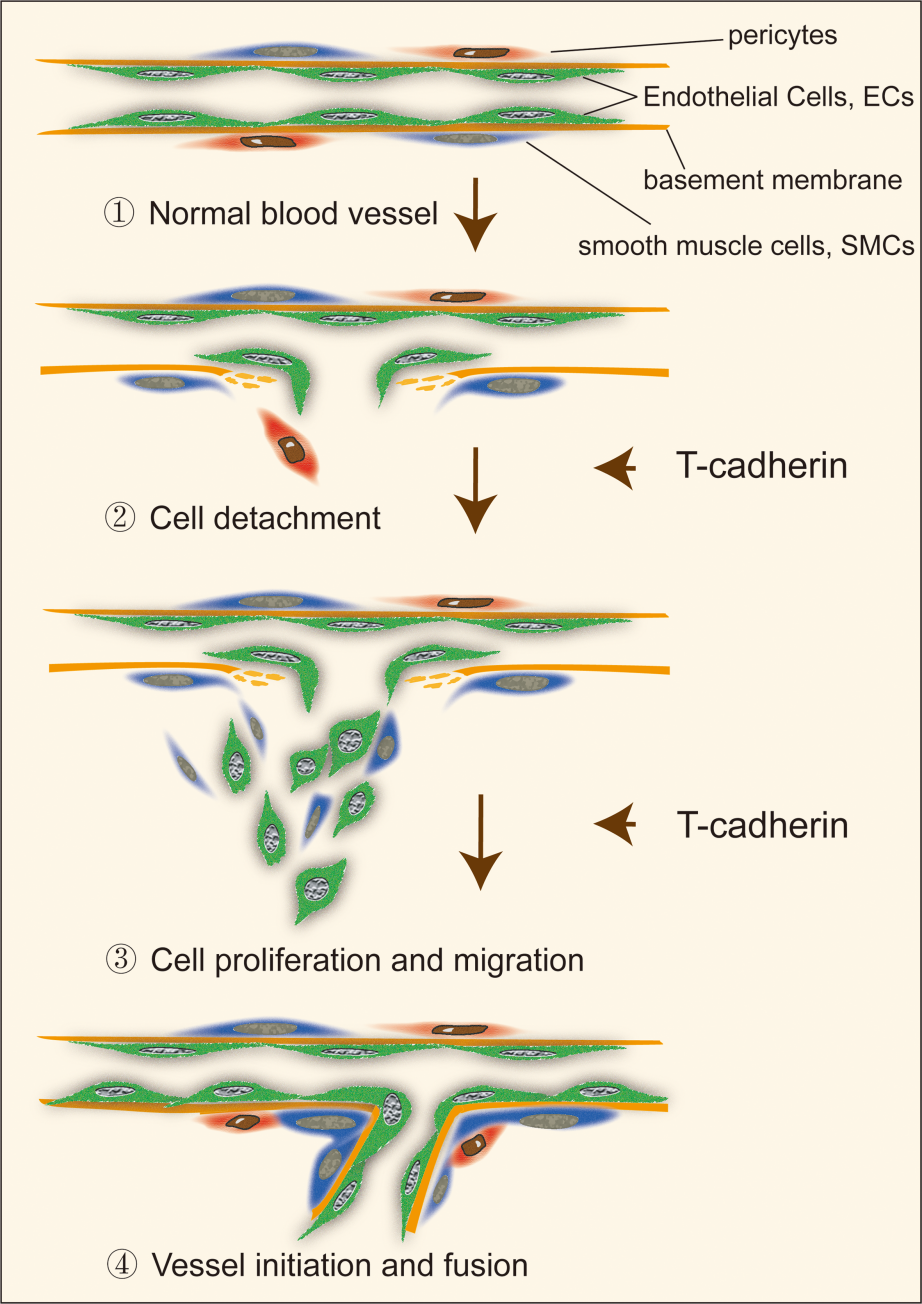
The role of T-cadherin in vascular cell detachment, migration and proliferation during capillary initiation.

T-cadherin homophilic ligation induced a capillary-like structure consisting of ECs in a 2D model and stimulated a sprout outgrowth in an EC spheroid model whereas an overexpressed T-cadherin in ECs by adenoviral infection increased the sprouting from spheroids (39). In this study, the effects of vascular endothelial growth factor (VEGF) on neovascularization were enhanced by T-cadherin participation in mouse skeletal muscle *in vivo* (40). However, angiogenesis induced by T-cadherin was not eliminated by inhibition of the VEGF receptor. T-cadherin induced sprouting in both the absence and presence of VEGF, yet it did not trigger neovascularization in the absence of VEGF, suggesting a complex role of T-cadherin in capillary initiation that may be independent of VEGF (39). However, another study investigated the incubation of recombinant N-terminal EC1 domain of T-cadherin in stroma, which is crucial for intercellular recognition and adhesion (41), inhibited endothelial capillary growth *in vitro* and had no effects on endothelial cell proliferation, adhesion or apoptotic induction (42). The contrary results of T-cadherin in regulation of capillary initiation may be attributed to the different tumor cells used in the above models, or the variable roles of T-cadherin in different tumor angiogenesis mentioned above. It is worth noting that T-cadherin, located in the tumor microenvironment, seems to have a more significant effect on tumor capillary initiation than other mechanisms. This effect on the tumor microenvironment, in turn, participates in tumor angiogenesis (**Figure 2**).

## 4 The association between T-cadherin expression and tumor vessels

In contrast to normal vascular structures, the vascular network of malignant tumors exhibits a disorganized and non-hierarchical arrangement, lacking the typical progression from arterioles to capillaries to venules. Malignant tumor vessels possess uneven distribution of the basal membrane, large differences in vessel caliber size, partially dissociated pericytes and SMCs from ECs and basal membrane (43, 44). Current studies have highlighted that a low or insufficient level of T-cadherin expression in a variety of tumor cells is often closely correlated with the malignant features, as observed in breast cancer, colorectal cancer, endometrial cancer, bladder cancer, melanoma, and squamous cell cancer (45–50). In contrast, high levels of T-cadherin expression have been detected in osteosarcoma, basal cell carcinoma and hepatocellular carcinoma (14, 51, 52). These contrasting findings strongly suggest a correlation between T-cadherin and tumor growth, indicating that the effects of T-cadherin on cell behavior can vary significantly across different cancer types (14, 53).

### 4.1 Lung caner

Overexpression of T-cadherin in tumor vessel endothelial cells was first observed in a Lewis lung carcinoma lung metastasis model (22). Increased expression of T-cadherin was observed in tumor penetrating vessels, while little or no T-cadherin was detected in tumor cells. Another study showed that T-cadherin was absent in 43% of 35 NSCLC tumors but present in all adjacent nonmalignant lung tissue (54). Aberrant promoter methylation may be an important mechanism underlying the low expression or inactivation of T-cadherin in lung cancer. However, there was no significant correlation between hypermethylation of T-cadherin promoter and clinicopathological features, smoking status, clinical stages, or EGFR (epidermal growth factor receptor) mutation status (55, 56). In addition, poorly differentiated NSCLC (non-small cell lung cancer) shows higher levels of T-cadherin promoter hypermethylation than moderately or highly differentiated NSCLC. furthermore, NSCLC patients without T-cadherin hypermethylation have longer overall survival than those with T-cadherin hypermethylation (56). Thus, T-cadherin may act as a tumor suppressor in lung cancer, and its inactivation could contribute to tumor progression and poor prognosis.

### 4.2 Breast cancer

Early studies indicated that CDH13 gene is frequently methylated in breast cancer, leading to downregulation of T-cadherin expression (57, 58). Consistent with clinical findings in lung cancer, negative expression of T-cadherin was significantly associated with poor prognosis in patients with axillary lymph node-positive breast cancer or triple-negative breast cancer (59–61). In addition, DNA polymerase β (Pol β) upregulated T-cadherin expression by promoting T-cadherin promoter DNA demethylation, which in turn inhibited tumor migration and invasion, further validating the suppressor role of T-cadherin in breast cancer (62). However, T-cadherin may not be a monofunctional tumor suppressor. Using a transgenic mouse model, researchers found that deletion of T-cadherin limited tumor formation, restrains neovascularization, causes hypoxia, and increases metastases to the lungs (63). Importantly, T-cadherin-deficient tumors exhibit reduced vascular density, enhanced apoptosis, and enlarged hypoxic and necrotic regions. When compared with that in wild-type tumors, poorly differentiated tumors were more prone to be observed in the T-cadherin-deficient condition, suggesting a crucial role of T-cadherin in supporting tumor growth. Considering the significant role of T-cadherin in vascularization, it appears that the contradictory conclusions may be attributed to the complex mechanisms underlying tumor angiogenesis or to the possibility that T-cadherin plays different roles at different stages of tumor angiogenesis.

### 4.3 Melanoma

CDH13 gene methylation is also prevalent in melanoma and has been investigated more extensively. A prior investigation evaluated the expression levels of T-cadherin in 40 human melanoma cell lines using RT-PCR (49). The study revealed that T-cadherin expression was significantly reduced or absent in 28 (70%) melanoma cell lines. However, the hypermethylation of CpG islands in the promoter region of the CDH13 gene is not the only mechanism responsible for the loss of T-cadherin. DNA demethylation and inhibition of histone deacetylase do not result in the re-expression of T-cadherin in melanoma cell lines. Additionally, certain transcriptional repressors, such as BRN2 in melanoma cells and ZEB1 in gallbladder cancer cells, inhibit the transcriptional activity of the CDH13 promoter (64). Specifically, in melanoma cells, the transcriptional repression of CDH13 promoter activity by BRN2 enhances their migratory and invasive capabilities (65).

Multiple studies found that T-cadherin expression in B16F10 melanoma cells remarkably reduced cell proliferation and invasion and promoted apoptosis, which may be associated with antagonizing the AKT/CREB/AP-1/FoxO3a signaling pathway. In mouse tumor models, smaller tumor masses and significantly decreased vascularization were observed in T-cadherin overexpressed group (66–68). In addition, melanoma cells with positive expression of T-cadherin were more sensitive to garcinol (a chemically synthesized polyisoprenylated benzophenone), which also demonstrated the inhibitory effect of T-cadherin on melanoma (69). However, the inconsistent influence of T-cadherin on tumor angiogenesis has been noted in other studies. First, expression of T-cadherin leads to increased invasive potential due to the upregulation of pro-oncogenic integrins, chemokines, adhesion molecules and extracellular matrix components. Furthermore, the overexpression of T-cadherin in HMEC-1 cells leads to the formation of a vascular network within melanoma in a 3D multicellular tumor spheroid model. The authors propose an intriguing hypothesis that T-cadherin, expressed by endothelial cells, may facilitate tumor angiogenesis exclusively within a pro-angiogenic microenvironment (70). Therefore, the role of T-cadherin in melanoma functions beyond the regulation of tumor cells, being closely associated with the mechanisms underlying tumor angiogenesis.

### 4.4 Squamous cell carcinoma

Early studies found that T-cadherin was specifically localized at the basal layer of normal epidermis but was downregulated in cutaneous squamous cell carcinoma (71, 72). In squamous cell carcinoma HSC-1 cells, overexpression of T-cadherin increased surface β1 integrin expression, inhibited β1 integrin internalization, and promoted β1 integrin-mediated cell-matrix adhesion, which was possibly associated with reduced phosphorylation at Tyr845 of EGFR. This suggests that T-cadherin acts as a negative regulator of epidermal tumorigenesis (73). However, in another study, the overexpression of T-cadherin in cutaneous squamous cell carcinoma A431 cells inhibited the adhesion between tumor cells and vascular ECs, whereas T-cadherin deficiency induced adhesion between A431 cells and ECs (74). It is possible that the expression or functional alterations of other intercellular adhesion molecules are due to T-cadherin loss. Moreover, more blood vessels were observed in T-cadherin overexpressed tumors than those in T-cadherin silenced tumors in cutaneous squamous cell carcinoma xenografts. Interestingly, the promoting effects of T-cadherin on tumor angiogenesis in this study appeared to be a direct contribution of T-cadherin towards creation of a proangiogenic microenvironment (75, 76). Although overexpression of T-cadherin in A431 cells did not affect the tumor cell proliferation *in vitro* and *in vivo*, the culture supernatant of T-cadherin overexpressed A431 cells still promoted the sprout outgrowth (75). Therefore, T-cadherin expression in the tumor microenvironment promotes angiogenesis rather than that expressed in tumor cells, aligning with the concept that T-cadherin regulates cell proliferation and morphological changes in vascular cells.

### 4.5 Hepatocellular carcinoma

Hepatocellular carcinoma (HCC) is generally believed to be a hypervascular tumor. Similar to the expression pattern of T-cadherin in other tumors, T-cadherin was underexpressed in hepatocellular carcinoma cells (26.5%, 13/49 cases) but was frequently (77.6%, 38/49 cases) overexpressed in tumor endothelial cells (77). Overexpression of T-cadherin induced G2/M cell cycle arrest, decreased cell proliferation, inhibited adherence-independent growth, and increased the sensitivity of HCC cells to TNFa-mediated apoptosis. They also found that T-cadherin significantly inhibited the activity of c-Jun, a key oncogene that is constitutively activated in hepatocellular carcinoma cells. T-cadherin was selectively expressed in intratumoral capillary endothelial cells, and the expression levels of T-cadherin was also positively correlated with tumor malignant progression (78). Specifically, the higher expression of T-cadherin was more likely detected in poorly differentiated tumor regions than in regions where the tumor cells were well differentiated, indicating that T-cadherin in sinusoidal vascular endothelial cells might be increasingly induced during the tumor progression (79).

### 4.6 Other tumor types

Decreased expression of T-cadherin was associated with the larger tumor size, surrounding tissue infiltration, lymph node metastasis and poor differentiation in gastric cancer (80, 81). Moreover, PI3K/AKT/mTOR signaling pathway, an important role in regulating angiogenesis both in normal tissues and in cancers, was reported to be involved in T-cadherin related tumorigenesis of human gastric cancer and cervical cancer, since sustained activation of AKT1 in endothelial cells has been shown to induce the formation of abnormal blood vessels, which was similar to the aberrations of tumor vessels (82–84).

## 5 T-cadherin as a receptor for LDL and adiponectin on vascular endothelium

Another important role of T-cadherin is to function as the receptor of low-density lipoproteins (LDL) and adiponectin. In the early 1990s, T-cadherin was purified and identified from human and rat aorta, as well as cultured SMCs, as a receptor of LDL (85). However, the mechanism underlying the specificity interaction of T-cadherin and LDL in tumor angiogenesis has not been studied in detail.

Adiponectin is an adipose tissue-derived homeostatic factor that is mainly secreted by white adipocytes, but can also be produced by skeletal muscles, cardiac myocytes and ECs (86, 87). In plasma, Adiponectin exists in a variety of complexes, including trimers (LMW, low molecular weight), hexamers (MMW) and high molecular weight multimers (HMW), exerting protective functions in insulin−sensitizing, anti−inflammation, anti−proliferation, anti−atherosclerotic action and tumor suppression of various tissues (87). It is believed that the HMW adiponectin is the metabolically active form of adiponectin (88). However, the function of adiponectin in blood vessel remains controversial, both protective and promotive effects on blood vessel growth are reported (63, 89, 90).

Except AdipoRs (adiponectin receptors), T-cadherin seems to be another major receptor for native adiponectin in serum with still largely unknown role in intracellular signaling. T-cadherin was co- localized with adiponectin in vascular endothelium, pericytes, and skeletal muscles (91). Early studies have demonstrated that binding of eukaryotic-expressed adiponectin to cell-surface T-cadherin is dependent on the hexameric and HMW form (92). Subsequent research by Kita et al. demonstrated that native adiponectin selectively binds to cells expressing T-cadherin, while no binding was observed in cells expressing AdipoR1. Furthermore, the knockdown of T-cadherin led to a marked reduction in adiponectin binding to these cells (93). Additionally, cardiovascular tissues lacking T-cadherin appear to be insensitive to adiponectin, despite with the consistently expressed AdipoR1/AdipoR2 (94). Adiponectin induces EC differentiation into capillary-like structures and stimulates blood vessel growth by promoting cross-talk between AMPK and Akt signaling, which may be the same as the function of T-cadherin on ECs (95). Furthermore, T-cadherin expressed by tumor endothelial cells inhibits cell apoptosis during tumor angiogenesis by activating NF-κB upon binding to hexamer and/or HMW adiponectin (79). Contrarily, another study found that adiponectin can significantly inhibit proliferation and migration of ECs via the activation of the caspase-mediated ECs apoptosis, and recombinant adiponectin potently impaired primary tumor growth that is associated with decreased neovascularization in mice (89). Using an ischemia-induced revascularization model, the impaired revascularization phenotype could be rescued by overexpression of adiponectin in APN-KO (adiponectin knockout) mice but not in mice that were lacking T-cadherin, suggesting an essential role of T-cadherin in mediating the proangiogenic activity of adiponectin. Supportively, the study also found that knockdown of T-cadherin prevented adiponectin induced migration and proliferation of cultured ECs (96). Similarly, in MMTV-PyV-mT mice, T-cadherin was able to sequester adiponectin to the vasculature in a T-cadherin-dependent manner while the plasma adiponectin level was dramatically increased in T-cadherin-deficient mice (63). Moreover, in the absence of T-cadherin, adiponectin appears to lose its location in the vasculature, suggesting that T-cadherin plays a crucial role in the regulatory mechanisms of vascular function involving adiponectin in both normal tissues and malignant breast cancer. In the light of these findings, although the interaction of adiponectin and T-cadherin has been well established, the role of this binding on tumor angiogenesis is still not clear. In addition, T-cadherin seems to be a membrane binding protein for adiponectin rather than a receptor that needs to be confirmed by further studies in the future.

## 6 Conclusion and perspective

**Figure 3:**
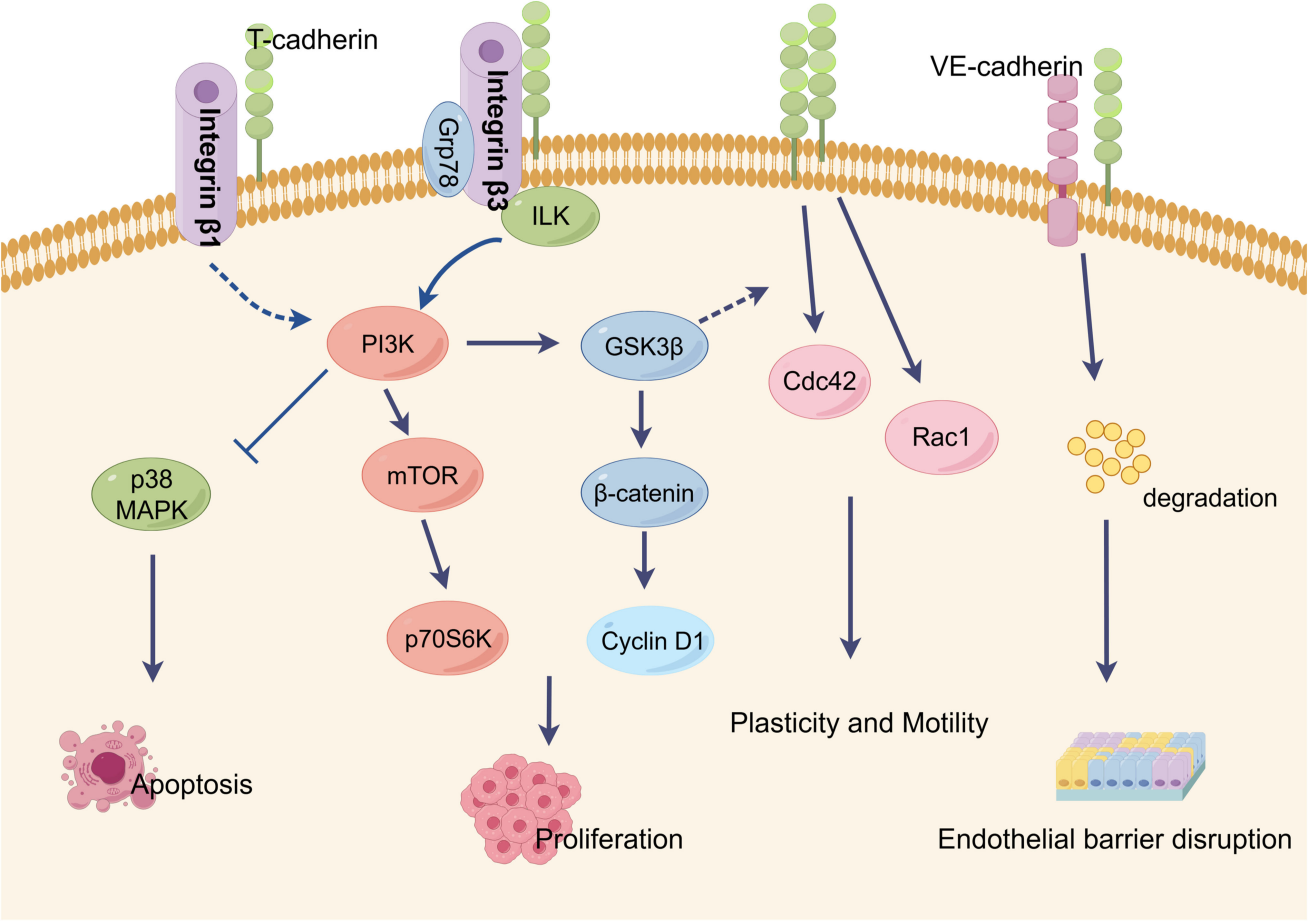
Characteristic T-cadherin-mediated intercellular signaling events and biological activities. T-cadherin lacks transmembrane and cytoplasmic domains and localizes in membrane lipid rafts through a glycosyl phosphatidylinositol (GPI). T-cadherin is thought to be a regulator of endothelial cell survival, proliferation, plasticity and motility, all of which are key processes in angiogenesis. By interactions with GRP78 or integrin-β3, T-cadherin stimulates cell proliferation and protects endothelial cells from apoptosis by activation of the PI3K/Akt pathway. T-cadherin also inhibits integrin-β1 internalization in squamous cell carcinoma cells. Homophilic activation of T-cadherin induced morphological changes toward promigratory phenotype via RhoA/ROCK and Rac pathways. T-cadherin is able to induce the degradation of VE-cadherin in lysosomes, resulting in the disruption of endothelial barrier function. This figure is drawn by Figdraw.

T-cadherin plays a crucial role in tumor angiogenesis by influencing blood vessel development in endothelial cells (ECs), smooth muscle cells (SMCs), and pericytes. Its function in angiogenesis relies on homophilic interactions or signal transductions as a membrane-binding protein (**Figure 3**). However, the specific effects of T-cadherin on vascular cell proliferation, detachment and migration have shown conflicting results in different studies. These contradictions may arise from the diverse nature of tumor types and limited expression of T-cadherin in tumor cells. While T-cadherin is not the primary initiator of angiogenesis, it acts as a modulator that requires initial vessel destabilization through angiogenic factors to facilitate phenotype conversion, proliferation, and survival of vascular cells (39, 97). T-cadherin’s presence in rapidly growing vessels suggests its role in guiding and directing tumor angiogenesis (98). Future research should focus on understanding the molecular mechanisms underlying T-cadherin’s effects on both normal and tumor blood vessels, particularly the intercellular signaling between tumor cells and vascular cells.
